# Thermal Dry Reforming of Bio-Oil Model Compounds

**DOI:** 10.1021/acs.energyfuels.6c01305

**Published:** 2026-06-23

**Authors:** Maria Virginia Manna, Davide Amato, Giovanni Fabozzi, Giovanni Battista Ariemma, Pino Sabia, Raffaele Ragucci, Mara de Joannon

**Affiliations:** Institute of Sciences and Technologies for Sustainable Energy and Mobilities, STEMS-CNR, Napoli 80125, Italy

## Abstract

This work presents an experimental investigation of the thermal dry reforming of hydrocarbons and oxygenated compounds representative of biomass pyrolysis bio-oils. Acetol, furfural, phenol, and syringol were used as model oxygenated compounds, while methane and propane were selected as benchmark hydrocarbons. Acetol and furfural were considered as pure feeds, while solid phenol and syringol were dissolved in acetol and furfural, respectively. Experiments were carried out in a tubular flow reactor at atmospheric pressure over a temperature range of 800–1350 K, using diluted stoichiometric feed/CO_2_ mixtures. Gas-phase products were analyzed online by micro-GC, while condensed liquids were characterized by GC–MS and Karl Fischer titration. The results show that thermal decomposition dominates at low and intermediate temperatures, whereas dry reforming becomes significant above approximately 1200 K for all oxygenated feeds and propane and above 1300 K for methane. For the acetol/phenol mixture, the presence of phenol does not alter the onset temperature of dry reforming but enhances hydrogen yield at high temperature and reduces CO_2_ conversion, while for the furfural/syringol blend, dry reforming becomes active above 1200 K; however, the contribution of thermal decomposition remains more persistent compared to the other feeds, as suggested by the H_2_/CO ratio remaining above the stoichiometric dry reforming value even at the highest temperatures investigated. CO_2_ conversion levels higher than 50% and up to 100% were achieved at higher temperatures (above 1300 K), depending on the feedstock. The hydrogen yield strongly depends on the chemical structure and varies significantly with temperature, reaching values close to 100% for acetol/phenol, furfural/syringol, and propane.

## Graphical abstract

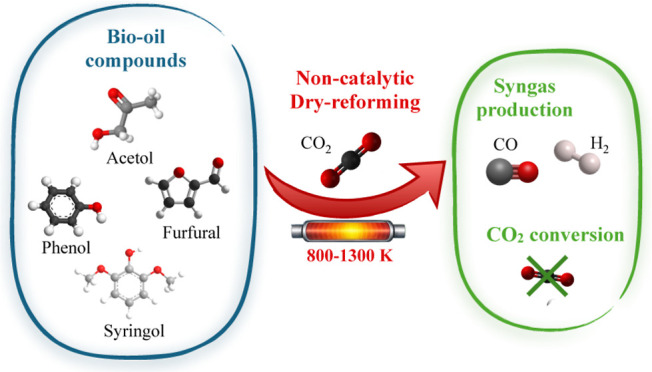

## 1 Introduction

The European and global tendency of recent years of moving toward renewable and sustainable energy sources has led to the consideration of alternative technologies.^1^ Apart from the standard renewable energy sources such as wind and solar energy, biomasses are getting more attention, given also the many possible valorization routes that they can undergo.^2^ A promising valorization treatment for biomass is pyrolysis, which can be considered carbon neutral under specific operational conditions and proper product utilization.^3^ Lignocellulosic biomass pyrolysis is a thermochemical process that converts the organic material of the biomass under an inert atmosphere, producing a solid carbonaceous product (biochar or char), a condensable fraction (bio-oil), and a gaseous phase (made principally by CO_2_, CO, H_2_, and CH_4_). Pyrolysis products have many possible utilizations, with bio-oil and gas being targeted recently for clean hydrogen and syngas production.^4–7^ In particular, if biomass pyrolysis is coupled with bio-oil dry reforming, it is possible to produce hydrogen while consuming CO_2_, moving the whole valorization chain from carbon neutral to carbon negative.^8,9^

In general, dry reforming is a process that converts hydrocarbon feeds into syngas following the (unbalanced) reaction

$$C_{x}H_{y}+CO_{2}=CO+H_{2}$$

For methane, this reaction is characterized by a high activation energy (247 kJ/mol) and requires high temperature (*T* > 1250 K) to achieve a good conversion efficiency while inhibiting undesired reactions.^10^ During thermal noncatalytic dry reforming, methane undergoes several reactions^11^ starting from CH_4_ pyrolysis to form ethylene (C_2_H_4_), first, and then acetylene (C_2_H_2_). Acetylene is the species that undergoes reforming with CO_2_, producing CO and H_2_. CO_2_ also reacts with the H_2_ derived from CH_4_ pyrolysis, producing water via the reverse water–gas shift (RWGS) reaction. The presence of water triggers the steam reforming reaction, which has an activation energy lower than that of dry reforming. When hydrocarbons more complex than CH_4_ are considered, different reactions can occur during dry reforming. There is no clear pattern in how the reaction mechanism changes as the number of carbon atoms grows. However, it is believed that the formation of acetylene through cracking/pyrolysis is a key step for the dry reforming of all C_2+_ hydrocarbons.^12^ The complexity of the reaction pathways can further increase when considering cyclical structures^13^ or ramified hydrocarbons.^14^

Pyrolysis bio-oil is a complex mixture^15,16^ of oxygenated compounds characterized by different chemical functionalities (e.g., ketones, aldehydes, carboxylic acids), aromatic compounds (e.g., monomers and oligomers of phenolic compounds, PAHs, and furans), water, and low concentrations of hydrocarbons. Given its nature, information about hydrocarbon reforming is not easily transferred to bio-oil. Moreover, pyrolysis bio-oil dry reforming is still a developing topic, thus, the available scientific literature is scarce. Applications of catalytic steam reforming,^17^ catalytic partial oxidation (POX) combined with steam reforming,^18^ and combined catalytic steam/dry reforming^19,20^ have been tested. The use of catalysts for reforming is common since it allows for better product yields while also lowering the operating temperature. Many types of metal-based catalysts have been considered, such as Ni/Al_2_O_3_ and Ni/La_2_O_3_ catalysts,^9^ Pt, Pd, Rh supported on alumina and ceria/zirconia catalysts,^21^ and biochar-supported catalysts such as Ni-biochar.^4^ However, the use of catalysts poses two main issues: on one hand, metal-based catalysts often require critical raw materials, which are subject to recent European and global regulatory policies.^22^ On the other hand, catalytic processes suffer from catalyst deactivation due to phenomena such as sintering and coke formation and deposition.^23^ Catalyst deactivation is particularly relevant when bio-oils or heavy hydrocarbon mixtures are considered as feedstocks due to their higher tendency to form coke.^24^ In this regard, noncatalytic reforming is an appealing solution for the valorization of pyrolysis bio-oil providing new pathways to improve the yield of valuable gases and mitigate the environmental impact of CO_2_ emissions while avoiding the depletion of critical raw materials. This is advantageous especially when advanced combustion processes, such as MILD combustion,^25^ can be coupled with thermal dry-reforming to sustain it with low-calorific-value fuels. For example, this is the case where pyrolysis and dry reforming belong to the same process pipeline.^26^

Fundamental studies are required to identify the operating conditions ensuring significant feed conversion into syngas and a relevant degree of CO_2_ capture. Given the complexity of bio-oil, it is necessary to develop a methodical study using surrogate compounds to gain insight into the reaction mechanism and the optimal operating conditions for noncatalytic dry reforming. The use of bio-oil model compounds is a consolidated practice, and several representative compounds have been tested in catalytic reforming processes either in blends^27,28^ or as pure compounds.^29–32^

In the present work, four compounds commonly found in bio-oil derived from the pyrolysis of lignocellulosic biomass have been chosen as model compounds, namely, acetol (C_3_H_6_O_2_),^33^ furfural (C_5_H_4_O_2_),^15^ phenol (C_6_H_6_O),^34^ and syringol (C_8_H_10_O_3_).^35^ These compounds are the simplest representatives of bio-oil compound classes: ketones, furans, phenols, and syringols, respectively. Pure model compounds and mixtures of them were used as feeds for dry reforming experiments, exploring the effect of temperature on the system evolution in the range 800–1300 K. Methane (CH_4_) and propane (C_3_H_8_) were also considered as benchmark model hydrocarbons and were analyzed under the same operational conditions.

## 2 Methodology

The experimental tests were carried out in a stainless-steel helical tubular flow reactor, with an internal diameter of 0.01 m and a total length of 5.6 m. The reactor is located between two semicylindrical electrically heated ovens, and the heated length of the tube is about 5.3 m. The temperature inside the ovens, in the middle of the spiral, is monitored using five type-N thermocouples, equidistributed over a height of 40 cm. Air is insufflated within the ovens to homogenize the temperature. The averaged value of the five thermocouples was chosen as the reference temperature (*T*_ref_) for the experimental tests. Temperatures within the tubular reactor are measured by two thermocouples, located at the inlet and outlet of the heated section, respectively. The maximum temperature difference between the upper and lower thermocouples in the oven was 50 K, while the difference between the internal reactor thermocouple and the nearest external one did not exceed 10 K, suggesting negligible heat losses along the reactor. The gas flow speed was fixed at 1.7 m/s to have a residence time in the heated section of the reactor of about 3.1 s. The operating velocity was selected to ensure laminar flow throughout the reactor under all experimental conditions. CFD simulations performed at 900 and 1350 K, using the experimental gas mixture modeled as an ideal gas with temperature-dependent transport properties, showed velocity profiles with a maximum deviation from the ideal parabolic profile of approximately 30% at 900 K, reducing to ∼17% at 1350 K, confirming that the flow approaches the laminar ideal flow more closely at higher temperatures, where the chemistry of interest for dry reforming occurs. The detailed comparison and the calculated residence time distribution are reported in the Supporting Information (Figures S1 and S2).

The exhaust gases are fed to a condenser and cooled to 283 K. The condensed liquid phase is then collected and characterized offline, while the dry gases are analyzed by online microgas chromatography (Agilent 990 Micro GC). The gas analysis system was calibrated by using specific gas mixtures containing known concentrations of H_2_, O_2_, N_2_, CO, CH_4_, CO_2_, and C_2_.

The experiments were repeated twice for each feed, and the estimated experimental error varies between 10%–15% of the reported concentrations.

For each test, the collected liquid was analyzed following the methodology used for bio-oil characterization in previous studies.^36^ Briefly, the condensed liquid phases were analyzed for water concentration by carrying out Karl Fischer (KF) titration using a Metrohm Omnis KF Titrator. The condensed liquid samples were also analyzed with an Agilent 7890A gas chromatography system (GC) coupled with a mass spectrometer (MS) 5975C-VLMSD (GC–MS). The detailed description of the analysis method is reported in previous work.^36^ Compound identification is carried out by matching the obtained spectra with the National Institute of Standards and Technology (NIST) library. The results are reported in terms of the area percentage of the identified compounds with respect to the total area of revealed compounds. The compounds with a quality match of less than 80 were not considered in the characterization, but they were still included in the total area calculation.

As reported in the Introduction section, the compounds selected to be dry reformed are acetol (C_3_H_6_O_2_), furfural (C_5_H_4_O_2_), syringol (C_8_H_10_O_3_), and phenol (C_6_H_6_O). These compounds have varied physical properties that make their feeding into experimental systems for dry reforming quite challenging. The components that are liquid at room temperature (i.e., acetol and furfural) are fed to a Coriolis mass flow controller through a pressurized vessel to be prevaporized by a controlled evaporation and mixing system with N_2_ as carrier. However, this approach is not feasible for components, such as syringol and phenol, that have melting temperatures above room temperature, at 318 and 330 K, respectively.

In such cases, the adopted strategy consists of dissolving the solid components in a suitable liquid carrier to enable a liquid feeding. The effectiveness of this method depends on the solubility of the solid compound in the selected solvent and on the absence of chemical interactions between the two components, which must remain chemically inert in solution. Both furfural and acetol were considered as solvents, and the solution stability was evaluated by GC–MS analysis on the day of preparation and after 5 days. On the basis of the outcomes of several analyses, the mixtures selected for dry reforming studies were furfural-syringol (80–20 wt %) and acetol-phenol (80–20 wt %).

For comparison, the dry reforming of two hydrocarbons (methane and propane) was investigated under the same operating conditions.

The experiments were performed for stoichiometric bio-oil component feed/CO_2_ mixtures with stoichiometry defined according to the dry reforming reaction for each compound or solution considered. The mixtures were diluted with N_2_ at 92% vol. The mixture composition for each feed is reported in Table 1 along with the corresponding dry reforming reaction.

**Table 1:** List of Investigated Feeds, Corresponding Overall Reforming Reactions, and Inlet Mixture Compositions (%Vol)

feed	dry reforming reaction	mixture composition (%vol) feed-CO_2_–N_2_
acetol	C_3_H_6_O_2_ + CO_2_ = 4CO + 3H_2_	4.00–4.00–92.0
furfural	C_5_H_4_O_2_ + 3CO_2_ = 8CO + 2H_2_	2.00–6.00–92.0
phenol	C_6_H_5_OH + 5CO_2_ = 11CO + 3H_2_	-
syringol	C_8_H_10_O_3_ + 5CO_2_ = 13CO + 5H_2_	-
13%syringol-87%furfural	0.13C_8_H_10_O_3_ + 0.87C_5_H_4_O_2_ + 3.27CO_2_ = 8.67CO + 2.40H_2_	1.87–6.13–92.0
16%phenol-84%acetol	0.16C_6_H_5_OH + 0.84C_3_H_6_O_2_ + 1.66CO_2_ = 5.14CO + 2.99H_2_	3.00–5.00–92.0
propane	C_3_H_8_ + 3CO_2_ = 6CO + 4H_2_	2.00–6.00–92.0
methane	CH_4_ + CO_2_ = 2CO + 2H_2_	4.00–4.00–92.0

To further investigate the experimental data, numerical simulations were performed as a support. The kinetic mechanism used for the calculation is the one developed by Debiagi et al.^37^ for modeling biomass pyrolysis, gasification, and combustion as it includes all the chemicals considered in this study.

The aim of the simulations was to evaluate the evolution of the H_2_/CO ratio, a key parameter for the dry reforming process, for the selected compounds. The ratio was evaluated for stoichiometric mixtures under isothermal equilibrium conditions with CHEMKIN PRO,^38^ at pressure, temperature, and mixture composition consistent with the operating conditions. In addition, chemical kinetic analyses were carried out to identify the controlling pathways and to understand how the molecular structure can affect the global reactivity and, specifically, the activation of the dry reforming reaction.

## 3 Experimental Results

The following sections present the experimental results for the different feeds. The composition of the gaseous products is discussed in terms of concentrations of CH_4_, CO_2_, H_2_, and CO as a function of the reference temperature. For the liquid products, the concentrations (*x*) are reported on a dry basis (*x*_dry_), namely, the actual concentration (*x*_actual_) is affected by the condensation of unreacted feed and any formed liquid (*x*_liquid_species_), according to the equation

$$x_{dry}=\frac{x_{actual}}{\left(1-x_{liquid\text{\_}species}\right)}$$

The condensed products are collected for acetol and furfural and for the mixture acetol-phenol. Three sampling temperature intervals were considered, specifically 823–923 K, 923–1073 K, and >1073 K. Generally, it has been observed that the higher the reaction temperature, the lower the quantity of collected liquid. There is a temperature, which depends on the considered feed, above which liquid is not collected anymore.

### 3.1 Acetol and Acetol/Phenol Mixture

For acetol (Figure 1), the concentrations of H_2_ and CO increase monotonically for temperatures above 850 K. In particular, the slope of the CO concentration profile becomes steeper for temperatures higher than 1250 K. Methane is also formed over the investigated temperature range and shows a nonmonotonic behavior, with a maximum around 1050 K. Other hydrocarbons, such as C_2_H_2_ and C_2_H_4_, are also detected, although at relatively low concentrations (reported in the Supporting Information). Regarding CO_2_, its concentration slightly decreases in the temperature range of 850–1200 K. This behavior can be mainly attributed to changes in the total number of moles and the condensation of water and any heavier compounds. For temperatures above 1200 K, the CO_2_ concentration decreases sharply, reaching complete consumption at 1350 K. The observed trends suggest that at temperatures below 1200 K, acetol decomposition is the dominant process, leading to the formation of CO, H_2_, CH_4_, and C_*x*_H_*y*_ species, while CO_2_ remains largely unaffected. The dry reforming process becomes significant above 1200 K, as indicated by the rapid CO_2_ consumption, the increased slope of the CO and H_2_ concentration profiles, and the decrease in CH_4_ and other hydrocarbon concentrations.

**Figure 1:**
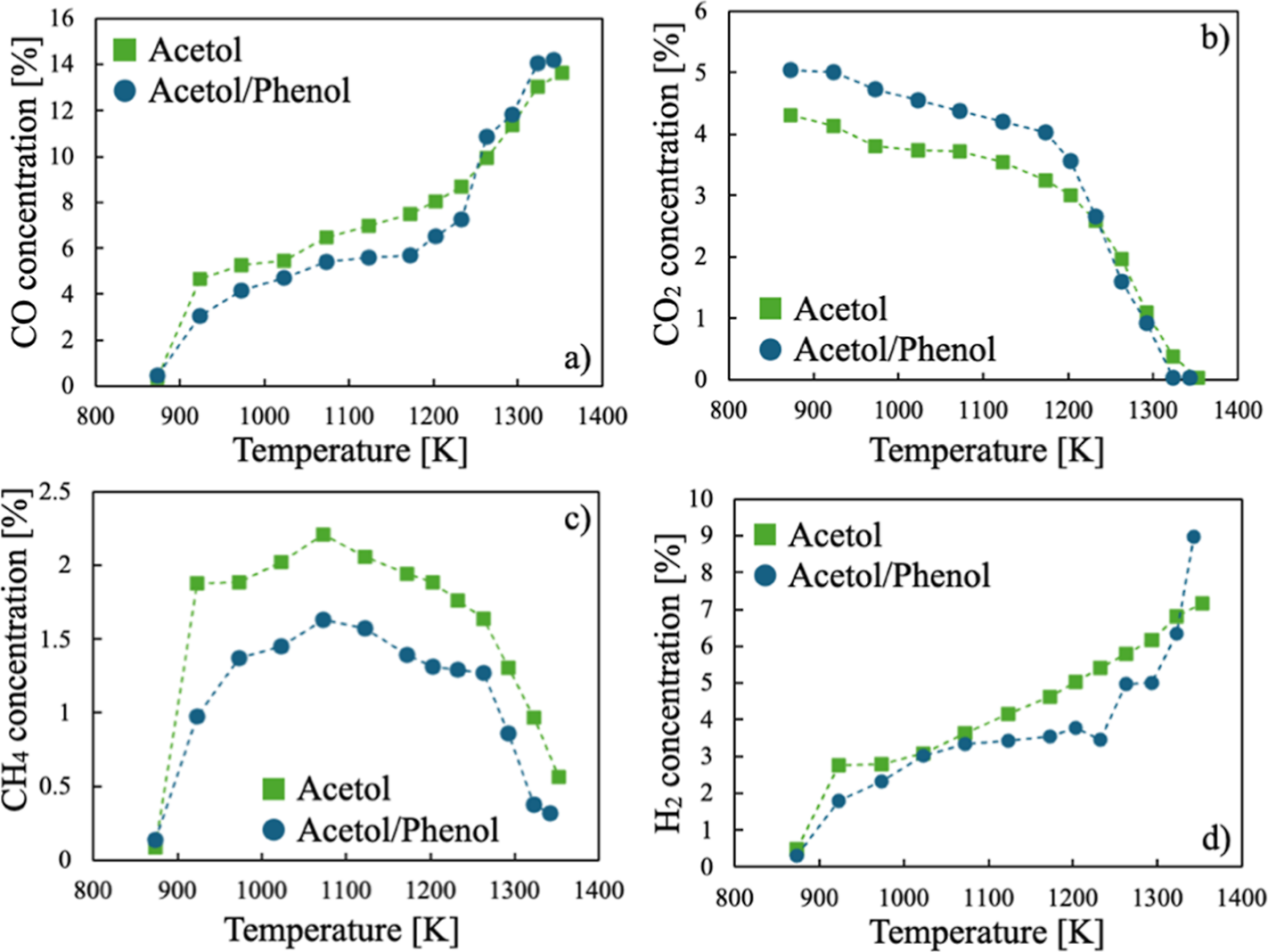
CO, H_2_, CO_2_, and CH_4_ concentrations for acetol and acetol/phenol mixture as a function of the temperature. Experimental error 10–15%.

The acetol-phenol mixture exhibits a similar overall behavior, although with different concentrations of H_2_, CO, and CH_4_ due to the different elemental compositions and stoichiometries of the fuel mixture. In this case, the onset of the dry reforming process is even more pronounced, as evidenced by the increased slopes of both the CO and H_2_ concentration profiles. At complete CO_2_ conversion, a higher production of H_2_ and CO is observed. The comparison between acetol and the acetol-phenol mixture indicates that the presence of phenol does not influence the onset temperature of the dry reforming process significantly.

The composition of the liquids condensed during acetol reforming does not change drastically, as reported in Figure 2A. A more complete characterization is reported in Table S1 of the Supporting Information. As the reaction temperature increases, the amount of unreacted acetol slightly decreases from 92.3% to 87.2% of the total chromatogram area. At low temperature (823–923 K), the other identified compound is 4-hydroxy-4-methyl-2-pentanone, accounting for 2.4% of the total area. At temperatures in the range 923–1073 K, acetoin is also present in the condensed liquids, and 4-hydroxy-4-methyl-2-pentanone area decreases to 1.1%. Both these compounds retain the same chemical functionality as acetol (=O and –OH groups) while increasing the length of the hydrocarbon base chain, passing from 3 carbon atoms for acetol to 4 and 6 for acetoin and 4-hydroxy-4-methyl-2-pentanone, respectively. At temperatures above 1073 K, the presence of acetoin increases reaching roughly 4%. Neither acetoin nor 4-hydroxy-4-methyl-2-pentanone is reported to be produced by the direct reaction of acetol.^39^ However, Wang et al.^40^ postulated a formation mechanism for acetoin that starts from acetol decomposition intermediates. It is likely that 4-hydroxy-4-methyl-2-pentanone is formed through an analogous mechanism.

**Figure 2:**
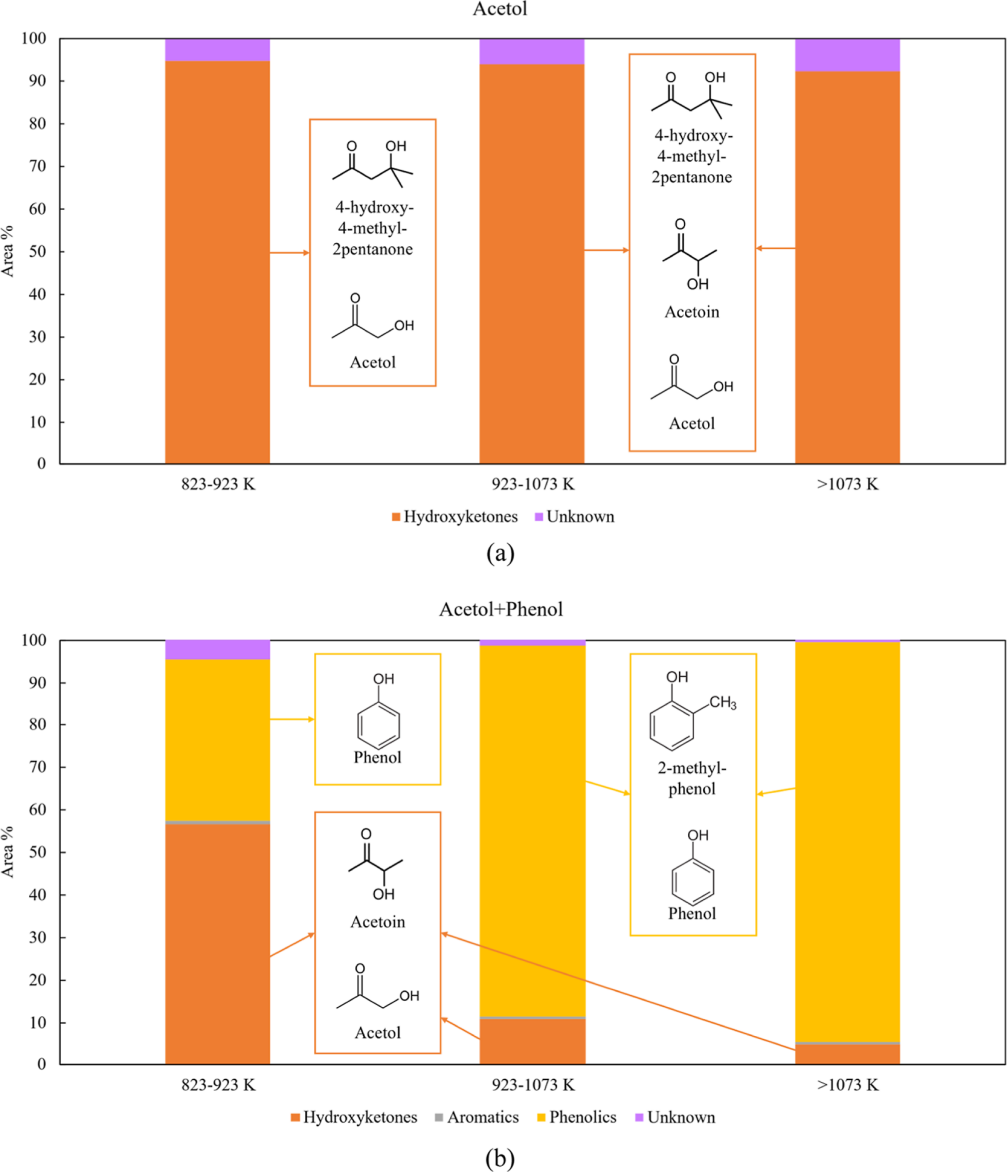
GC–MS analysis of the liquid products collected during the reforming of acetol (a) and acetol-phenol mixture (b).

Analysis of the condensed liquid through filtration revealed no solid particles at any investigated temperatures.

The water content (Table S1) of the collected liquids increases with the temperature from 5.3 to 11.9 wt %. Water formation during dry reforming of acetol could be attributed to the cleavage of the hydroxy group during thermal decomposition, which is a more favored reaction than RWGS for water formation, in particular at lower temperatures. No significant amount of liquid was collected above 1200 K.

The addition of phenol to acetol increases the complexity of the collected liquids (Figure 2b), whose detailed composition is reported in Table S1. In the temperature range 823–923 K, the main compounds are the unreacted reagents (acetol and phenol accounting for 54.8% and 37.8% of the total area, respectively) together with small amounts of the same products identified for pure acetol dry reforming (4-hydroxy-4-methyl-2-pentanone and acetoin). The presence of phenol anticipates the appearance of acetoin, which is detected at higher temperatures in the case of a pure acetol. Moreover, traces of aromatics (benzene and pyrene, directly derived from phenol pyrolysis^41^) and phenol-derived esters (acetic acid phenyl ester) are found. As the reaction temperature increases, the amount of unreacted acetol decreases, reaching 9.0% at 923–1073 K and 4.1% over 1073 K. In contrast, the relative abundance of unreacted phenol greatly increases, reaching 88.4% of the total chromatogram area at temperatures higher than 1073 K. This indicates that as the reforming temperature increases, the amount of unreacted phenol greatly exceeds the amount of unreacted acetol. While no quantitative evaluation of the unreacted reagents was possible, the low amount of collected liquid at high temperatures suggests that both phenol and acetol are mostly converted into other products. Small quantities of species derived from phenol decomposition are detected as reforming temperature increases. Between 923 and 1073 K, substituted phenols such as p-cresol and 2-methyl-phenol are identified. A further increase in the reaction temperature causes an increase in their relative abundance, and other polycyclic aromatic hydrocarbons are also formed (e.g., naphthalene). Differently from what was observed for pure acetol, the percentage of both acetoin and 4-hydroxy-4-methyl-2-pentanone decreases as reforming temperature increases. The water concentration of the collected liquids (Table S1) follows the same increasing trend with temperature observed for pure acetol with slightly lower values, ranging from 4.9 to 10.0 wt %. The decrease in the water concentration with respect to pure acetol is due to the lower overall reactivity of the added phenol. Also in this case, the condensed phase collected at the reactor outlet is negligible for *T* > 1200 K.

### 3.2 Furfural and Furfural/Syringol Mixture

In the case of furfural (Figure 3), the CO and H_2_ concentrations increase with temperature up to about 1050 K, then the concentrations remain almost constant up to 1200 K, and finally a more pronounced increase is observed above approximately 1200 K, indicating the onset of the dry reforming reaction. Consistently, the CO_2_ concentration is nearly constant up to about 1200 K and then sharply decreases at higher temperatures. CH_4_ and C_2_H_4_ are also formed from the furfural decomposition.

**Figure 3:**
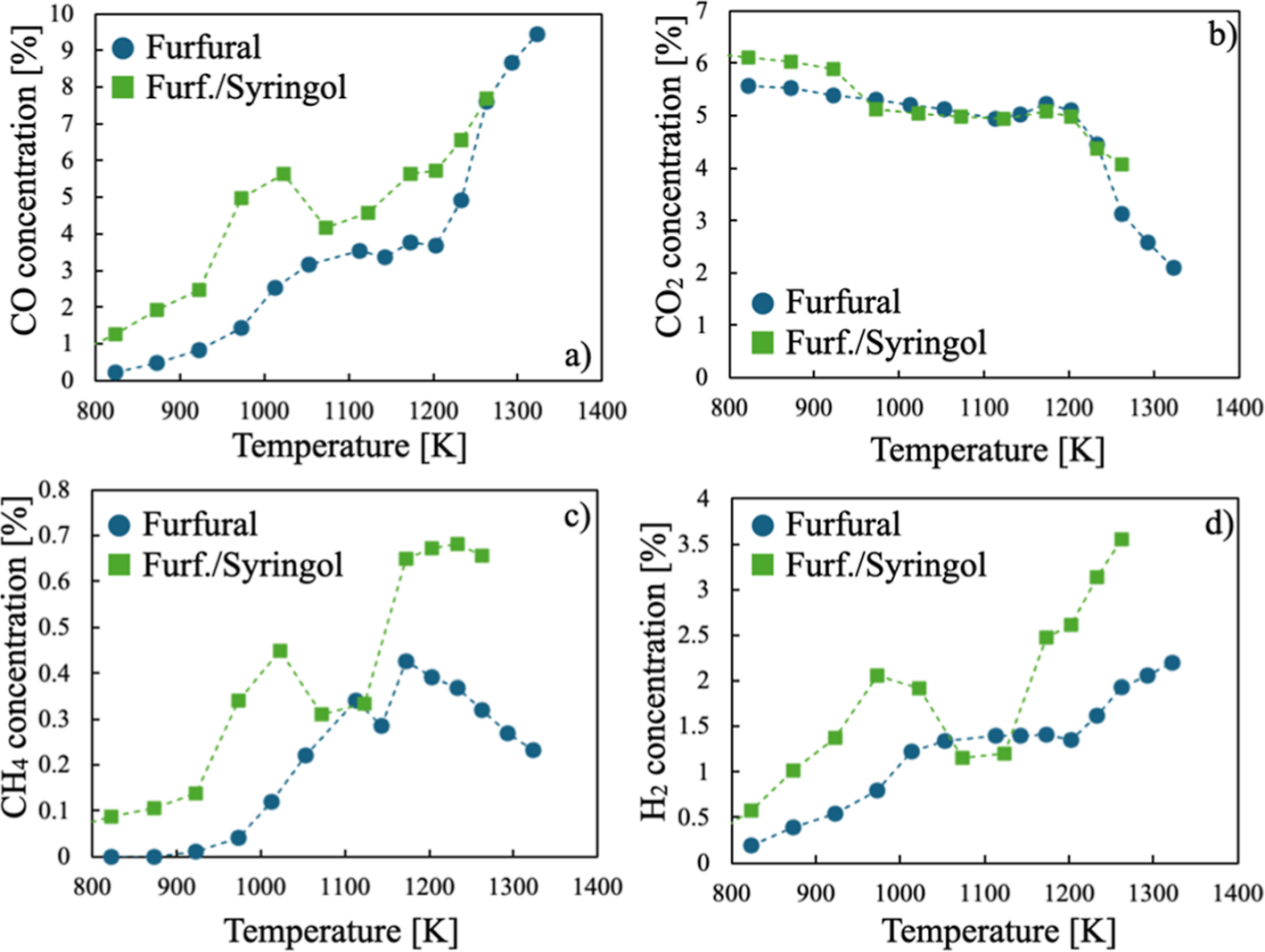
CO, H_2_, CO_2_, and CH_4_ concentrations for furfural and furfural/syringol mixture as a function of the temperature. Experimental error is 10–15%.

The methane concentration increases with temperature, reaching a maximum around 1200 K, followed by a gradual decrease at higher temperatures, where thermal decomposition starts to become marginal, with respect to dry reforming. C_2_H_4_ is produced in lower amounts but follows the same trend as CH_4_ (as reported in the Supporting Information). In particular, its concentration decreases to zero for *T* > 1250 K.

For the furfural-syringol mixture, higher concentrations of CO, H_2_, CH_4_, and C_2_H_4_ compared to those of pure furfural have been measured, consistent with the different elemental composition and stoichiometry of the mixture, while a lower CO_2_ conversion is observed at high temperatures. For both H_2_, CH_4_, and C_2_H_4_, the mixture shows nonmonotonic behavior, with a rapid decrease in the temperature range 1020–1120 K, suggesting a change in reaction pathways. This is also evident for CO concentration, since a rapid increase is observed between 950 and 1020 K, then its concentration decreases as for H_2_ and CH_4_ between 1020 and 1100 K, and finally increases again. The second stage of CO formation (*T* > 1100 K), however, is not coupled with a significant conversion of CO_2_ nor with a measurable decrease in methane concentration, both of which are typically indicative of the onset of dry reforming reactions. Therefore, these results suggest that the addition of syringol to furfural could introduce reaction pathways that compete with the activation of dry reforming reactions.

Liquid products were condensed during furfural reforming only in the two lower temperature intervals; at temperatures higher than 1073 K, no liquid formation was observed. Condensed liquids composition changes with reaction temperature, with increasing complexity as temperature increases, as reported in Figure 4 and, in detail, in Table S2 of the Supporting Information. In the temperature range 823–923 K, unreacted furfural is the main component of the liquid with 90% of the total area, followed by pyran-2-one with 4.2%. Pyran-2-one has the same molecular formula as furfural; however, the former has a pyran-like structure with a ketonic carbonyl group, while the latter is a furan ring with an aldehydic carbonyl group. This molecule, also called α-pyrone, is one of the main products of the thermal decomposition of furfural starting from 900 K, even if the exact reaction mechanism is still debated.^42^ Moreover, some small polycyclic aromatic hydrocarbons (PAHs) appear in the collected liquid, such as naphthalene, biphenylene, phenanthrene, and pyrene. When the reaction temperature increases in the range 923–1073 K, the unreacted furfural drops to 49% of the total area, and new species are identified in the condensed liquids. Pyran-2-one remains one of the most relevant compounds (11.4% area), together with small PAHs such as naphthalene and biphenylene (13.9 and 8.8% area, respectively). The presence of higher molecular weight PAHs increases (e.g., anthracene and pyrene with 4.4% and 2% area, respectively). Other aromatic-derived compounds are detected, such as substituted PAHs, (1-methyl-naphtalene and 2-methyl-naphtalene), phenols (phenol and p-cresol), and other complex polycyclic structures (indene). Finally, when reaction temperature exceeds 1073 K, no liquid products are collected.

**Figure 4:**
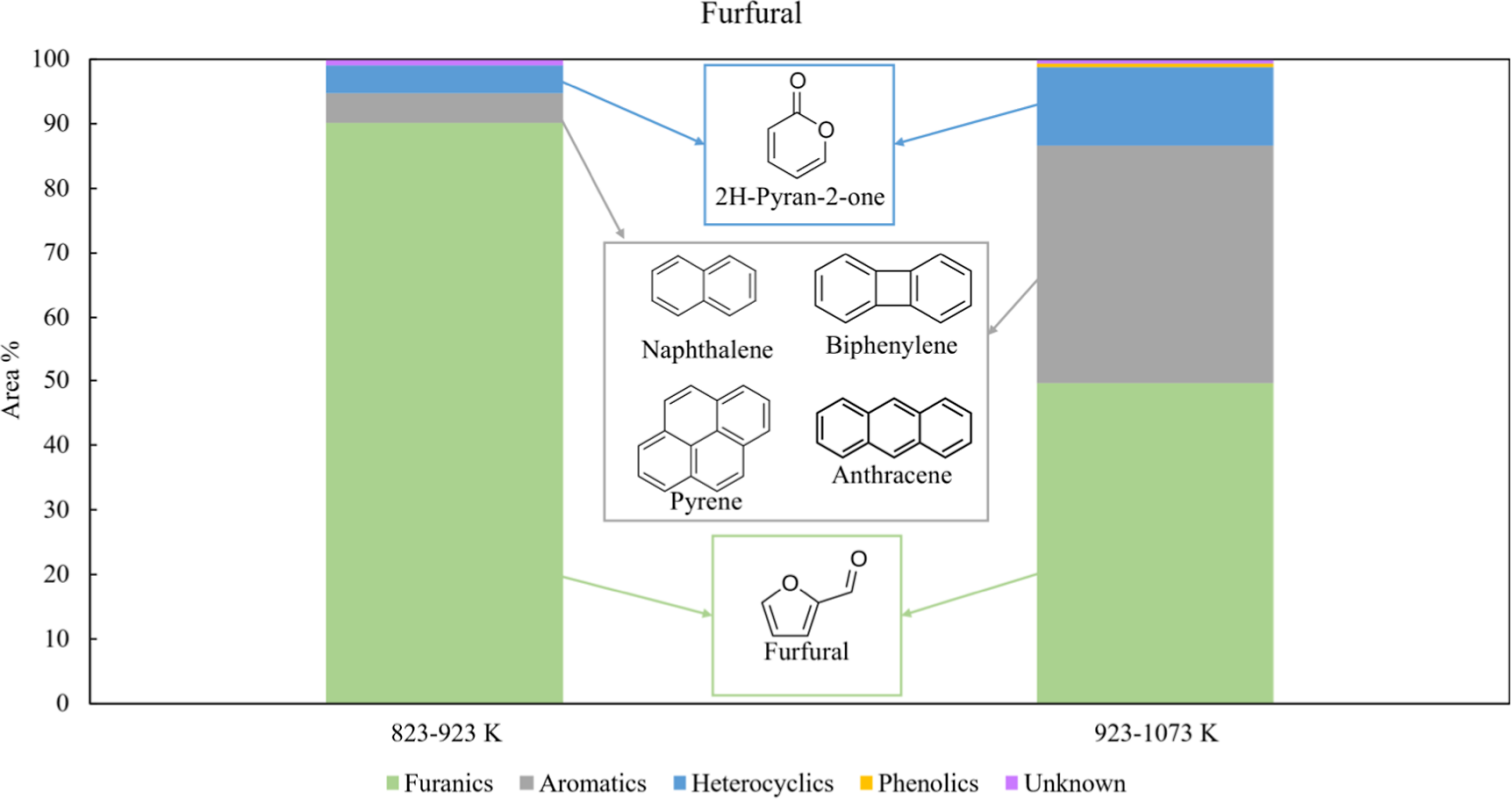
GC–MS of the liquid products collected during the reforming of furfural.

The filtration of the condensed liquid collected at the reactor outlet showed the absence of solid particles at all of the temperatures investigated.

Water concentration in the collected liquids (Table S2) slightly increases with reforming temperature, passing from 0.3 to 0.8 wt % in the whole considered temperature range. Water is not reported as a direct decomposition product of furfural;^42^ thus, its production must be attributed to other reactions such as RWGS.

### 3.3 Propane and Methane

The reactivity of propane (Figure 5) starts for temperature higher than 900 K, as shown by the monotonic increment of H_2_ and CH_4_, and formation of C_2_H_4_ (reported in the Supporting Information) while a significant CO formation is observed for *T* > 1150 K. Consistently, CO_2_ decreases and its full conversion occurs for *T* > 1250 K. Methane is significantly less reactive as its concentration starts decreasing for *T* > 1200 K, forming H_2_ and C_2_H_6_ (reported in the Supporting Information). CO and CO_2_ profiles suggest that the onset of the dry reforming occurs at about 1300 K; however, CO_2_ conversion is lower compared to propane at the same temperature.

**Figure 5:**
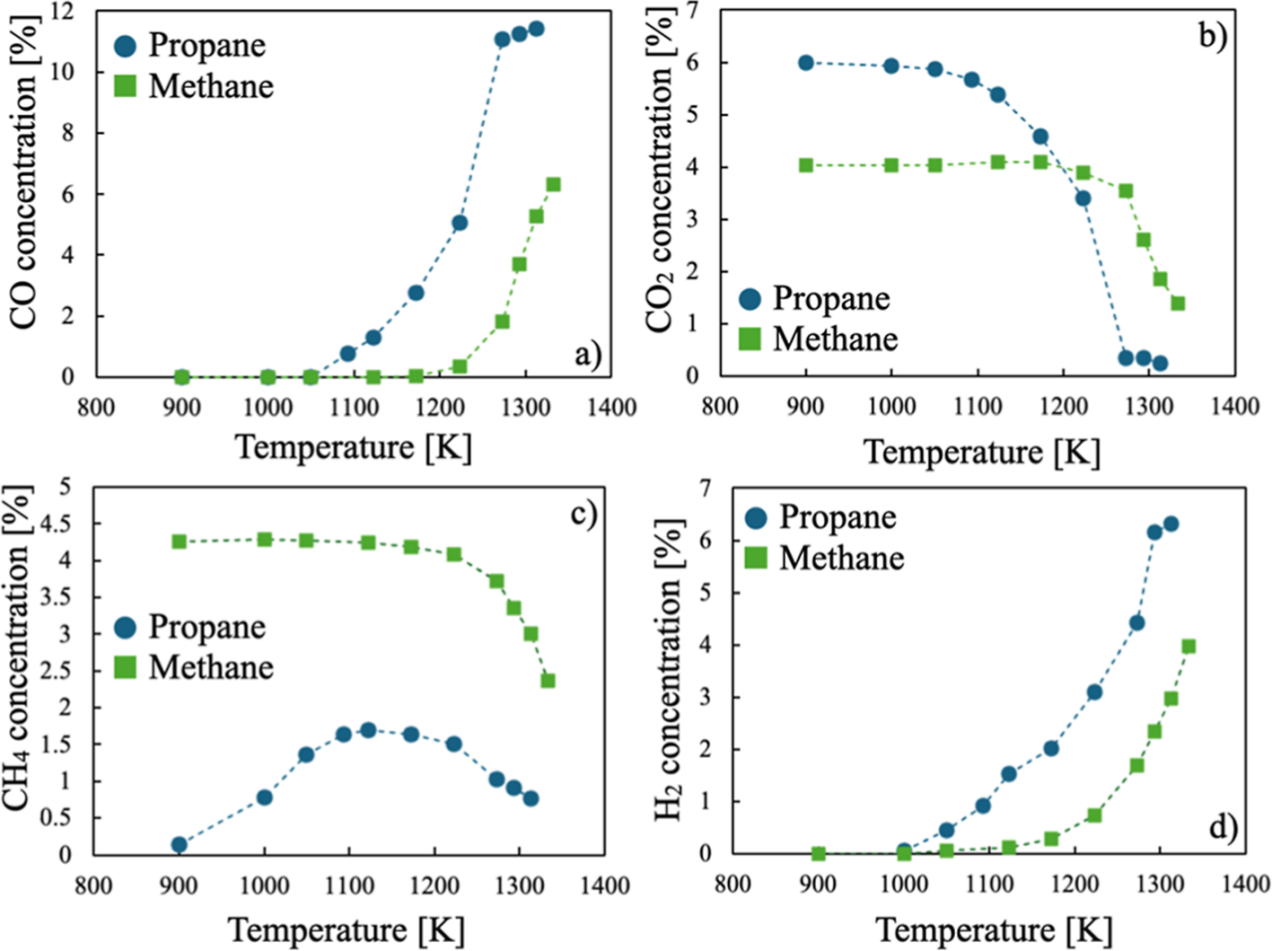
CO, H_2_, CO_2_, and CH_4_ concentrations for methane and propane as a function of the temperature. Experimental error is 10–15%.

### 3.4 Carbon and Hydrogen Elemental Balance

To assess the degree of elemental conservation and to support the discussion of the observed product distributions, carbon and hydrogen balances were evaluated for all four feeds, exploiting gas-phase measurements. The balances were calculated on a molar flow rate basis, using the inert N_2_ flow as an internal reference to convert dry-basis volume fractions into absolute molar flows. The carbon inlet flow was defined as the sum of the carbon contributed by the feed and the inlet CO_2_. The carbon outlet was computed as the sum of the C atoms carried out by all measured gas-phase species: CO, CO_2_, CH_4_, C_2_H_2_, C_2_H_4_, and C_2_H_6_. Analogously, the hydrogen inlet was defined from the H content of the feed molecule and the hydrogen outlet from H_2_, CH_4_, C_2_H_2_, C_2_H_4_, and C_2_H_6_. The average resulting balances, expressed as the ratio of the outlet to inlet elemental molar flow (%), are summarized in Table 2.

**Table 2:** Carbon and Hydrogen Elemental for the Investigated Feeds at Selected Temperature Interval^a^

feed	T interval	C balance (%)	H balance (%)
acetol	823–923 K	54	36
	923–1073 K	88	78
	1073–1200 K	98	92
	>1200 K	100	98
acetol/phenol	823–923 K	52	29
	923–1073 K	82	81
	1073–1200 K	85	89
	>1200 K	100	98
furfural	823–923 K	39	11
	923–1073 K	52	41
	1073–1200 K	62	62
	>1200 K	75	93
furfural/syringol	823–923 K	53	32
	923–1073 K	71	67
	1073–1200 K	73	75
	>1200 K	85	98

a Values represent the mean over the data points in each interval.

At low temperatures (823–923 K), the gas-phase carbon balance is close to approximately 39–54% depending on the feed, with the hydrogen balance even lower. This is consistent with the significant formation of condensed-phase products and condensed unreacted feed observed in this regime, which are not accounted for in the gas-phase balance. The larger gap in the H balance compared to C at low temperature can be explained by the condensation of water, quantified by Karl Fischer titration, which removes a significant fraction of hydrogen from the gas phase. These results further suggest that at low temperatures, the process is dominated by partial decomposition with substantial carbon and hydrogen partitioning into the condensed phase.

As the temperature increases through the 923–1073 K and the 1073–1200 K ranges, the C e H balances progressively improve for all feeds, reflecting the increased gas-phase conversion of the feed. For the acetol-based feeds, the balance approaches 96–98% (C) and 89–92% (H) already at 1073–1200 K, consistent with the low amount of liquid collected and the absence of solid particles in the condensed phase reported above. For the furfural-based feeds, the closure is slightly lower in the same range (60–73%). Above 1200 K, the gas-phase carbon and hydrogen balances for acetol and acetol/phenol reach almost 100%, while for the furfural and furfural/syringol mixtures, values are lower. The partial carbon deficit observed in the balance is attributable to the formation of heavy condensed-phase organics, which were found deposited on the reactor walls at the outlet section.

### 3.5 H_2_ Yield and H_2_/CO: Results and Discussion

H_2_ yield and H_2_/CO ratio represent the main targets of both the thermal decomposition and reforming reactions.

A high hydrogen yield of H_2_ indicates that such processes are proceeding efficiently toward the desired product, while side reactions, like polymerization or coke formation, are marginal. In addition, since the H_2_ absolute concentration is strongly affected by variations in total molar fraction and stoichiometry and dilution effects, expressing hydrogen as a yield, thus normalized to the amount of hydrogen in the feed, provides a more meaningful indication to assess the overall efficiency of the process and to compare different feed compositions.

Figure 6 shows the H_2_ yield as a function of the temperature for all of the selected components. In all of the cases, except for the furfural/syringol mixture, increasing temperature enhances the hydrogen yield, but the extent of this increase depends on the nature of the feed.

**Figure 6:**
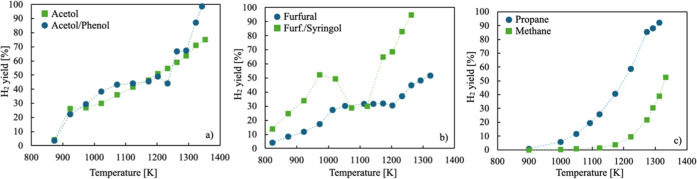
H_2_ yield as a function of the temperature for the selected components.

Acetol and acetol/phenol mixtures (Figure 6a) show very similar H_2_ yields. In general, the presence of phenol improves the results, especially at higher temperatures, where the acetol/phenol feed reaches very high H_2_ yields, approaching 100% at 1350 K.

In the case of furfural, the H_2_ yield is relatively low compared to acetol and acetol/phenol within the entire temperature range. Indeed, at the highest temperature, only 50% was achieved. When syringol is added, the hydrogen production is strongly enhanced. The furfural/syringol mixture shows a sharp increase in the H_2_ yield above about 1000 K and reaches considerably higher values at the highest temperatures, up to 95% at about 1270 K.

For the hydrocarbons (Figure 6c), H_2_ yields have a similar trend, but propane is more reactive within the considered temperature range, achieving high H_2_ yields, up to almost 100% at 1300 K, while at the same temperature, the H_2_ yield for methane is about 50%.

The H_2_/CO ratio is a key performance metric because it determines syngas suitability for downstream processes. From a reaction chemistry perspective, it reflects the system reactivity and how side reactions affect the process. For instance, in the case of methane,^43,44^ according to stoichiometry, the dry reforming reaction ideally gives H_2_/CO = 1. Side reactions like reverse water gas shift (RWGS) CO_2_ + H_2_ → CO + H_2_O and CO_2_ gasification C + CO_2_ = 2CO lower H_2_/CO, while methane cracking CH_4_ = C + 2H_2_ and steam reforming CH_4_ + H_2_O → CO + 3H_2_ can raise it. Certainly, the effective H_2_/CO is affected by stoichiometry (as reported in Table 1, with respect to dry reforming reactions) and operating conditions, like pressure, temperature, and feed/CO_2_ in the inlet.

In order to evaluate how far the actual H_2_/CO for each component is from the equilibrium value, thermodynamic-equilibrium calculations were performed at the same temperature, pressure, and feed composition and compared with the experimental results and the stoichiometric H_2_/CO based on the corresponding dry reforming reaction (Figure 7). Furthermore, equilibrium calculations were carried out at the same pressure and temperature but replacing CO_2_ with an equivalent amount of N_2_ to account for the effect of thermal decomposition.

**Figure 7:**
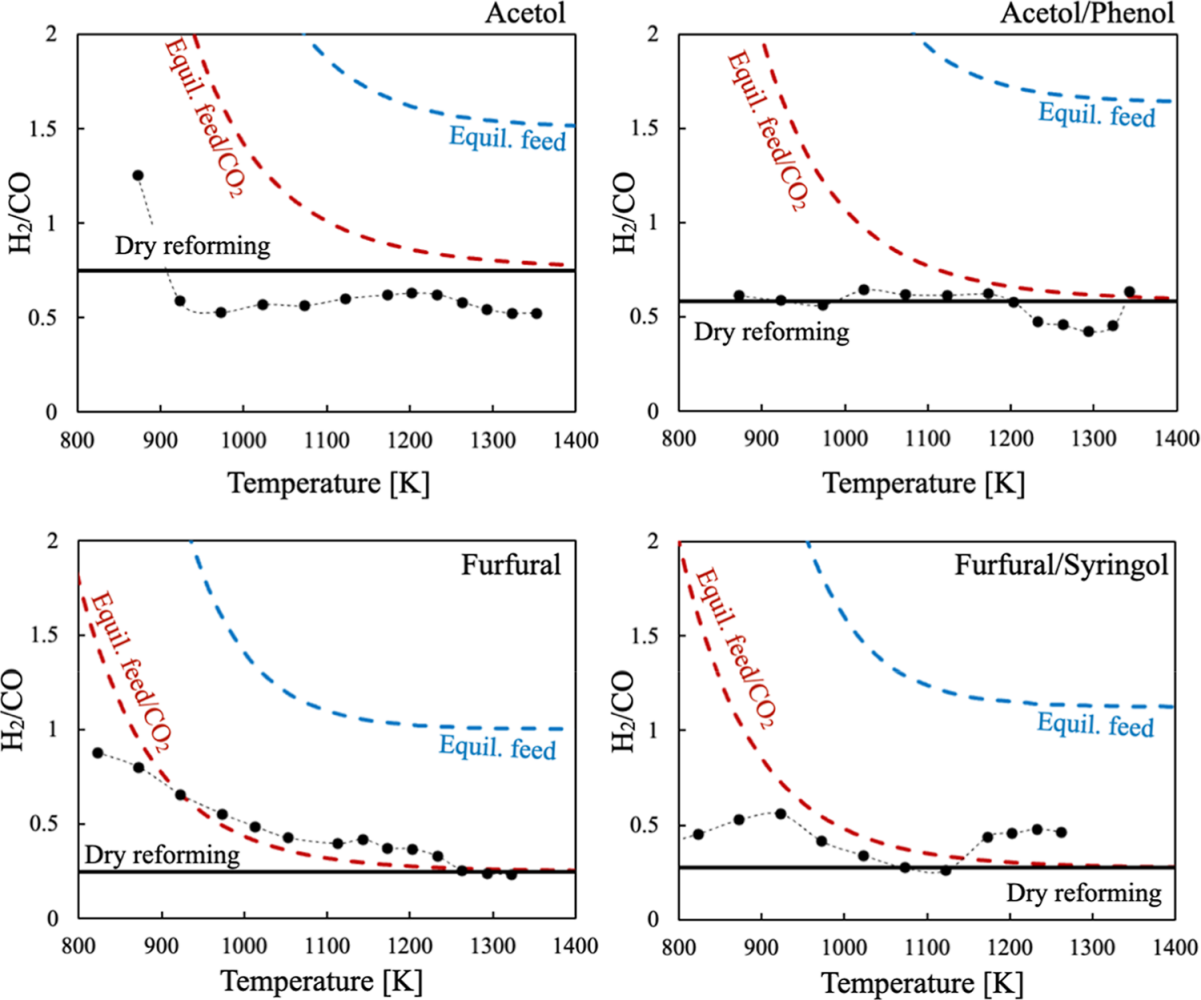
Experimental H_2_/CO (dots), stoichiometric H_2_/CO according to the dry reforming reaction (black line) and equilibrium calculation of H_2_/CO evaluated for each feed/CO_2_/N_2_ mixture (dashed red line) and for feed/N_2_ mixture (dashed black line) as a function of the temperature.

For all the components, the curves calculated under equilibrium conditions in the presence of CO_2_ (H_2_/CO_equilCO2_) decrease significantly with increasing temperature for all investigated compounds, progressively approaching the stoichiometric value expected for dry reforming (H_2_/CO_DR_).

In contrast, the equilibrium curves calculated in the absence of CO_2_ (H_2_/CO_equil_) display consistently higher H_2_/CO ratios, asymptotically approaching values close to unity or above at high temperatures.

For acetol, except for the point at 870 K, the experimental values are almost constant around 0.6 and remain below H_2_/CO_DR_, even for *T* > 1200 K where CO_2_ is effectively converted. For the acetol/phenol mixture, experimental H_2_/CO remains nearly constant up to 1200 K, and then it slightly decreases below H_2_/CO_DR_ and approaches again the H_2_/CO_DR_ value at 1350 K, where CO_2_ is fully consumed (Figure 1).

In the case of furfural, the experimental H_2_/CO is higher than H_2_/CO_DR_ and decreases with temperature, matching the theoretical value for *T* > 1250 K. When syringol is added to furfural, the experimental H_2_/CO exhibits a nonmonotonic trend. In particular, it remains above H_2_/CO_DR_ even at the highest temperatures, suggesting the dominant role of thermal decomposition over dry reforming reaction.

These results suggest that phenol barely influences the mixture reaction pathway, whereas the syringol decomposition pathway partially limits the contribution of dry reforming, shifting the onset of the reaction to higher temperatures.

### 3.6 Numerical Analysis and Reaction Pathways

To support the interpretation of the experimental results and identify the dominant reaction pathways for each feed, numerical simulations were performed using the kinetic mechanism developed by Debiagi et al. As a preliminary validation step, simulations were carried out using a laminar flow reactor (LFR) model implemented in CHEMKIN PRO,^38^ at the same pressure, residence time, and inlet mixture compositions as the experiments, under isothermal conditions. The simulated species profiles are reported in the Supporting Information (Figures S4 and S5). Although the model does not quantitatively reproduce the experimental concentration profiles, it somehow captures the main qualitative trends observed experimentally. In this context, it is worth highlighting that even though this kinetic scheme is, to the best of authors’ knowledge, the only one that simultaneously includes all the oxygenated species investigated in this work, it has been developed and validated for the description of gas-phase reactions of volatiles released during biomass pyrolysis and gasification, thus at temperatures, mixture compositions, and residences time different from those considered in the present work.

To identify the principal reaction pathways, a perfectly stirred reactor (PSR) model was then employed at the same residence time, pressure, and inlet compositions under isothermal conditions. The PSR configuration was chosen for kinetic analysis because it provides a homogeneous composition at each temperature, making the kinetic analysis straightforward to interpret without the implication of spatial gradients.

For each feed, the flux diagram analysis was performed at 1300 K, where the model suggests the onset of CO_2_ conversion, and at a lower temperature selected on the basis of the specific feed reactivity. Rate of production (ROP) analysis was performed for H_2_O and CO_2_ as a function of the temperature, covering the thermal decomposition regime and the dry reforming regime identified experimentally. The results are discussed below for each compound.

Simulations for acetol confirm that thermal decomposition dominates below approximately 1100 K, and acetol is fully converted for *T* > 1000 K. Acetol first undergoes decomposition to acetaldehyde (CH_3_CHO) and formaldehyde (CH_2_O), which reach peak concentrations around 850 K and are subsequently converted to CO, H_2_, and CH_4_ through a sequence of H-abstraction and decomposition steps involving CH_3_CO, CH_3_CHO, HCO, and CH_3_ radicals. Methane accumulates up to about 1100 K and decreases at higher temperatures when CH_4_ dehydrogenation becomes active. Ethylene and acetylene appear as products of the CH_3_ recombination pathway (2CH_3_(+M) = C_2_H_6_(+M), C_2_H_6_ = C_2_H_4_+H_2_). Benzene (C_6_H_6_) is predicted to form above 1100 K via CH_3_ recombination pathways, however, at very low concentration, with a peak of about 0.17% around 1300 K before decreasing. Soot precursor species (BIN1B) remain below 0.02% across the entire temperature range. These results confirm that coke and heavy aromatic formation are negligible for acetol under the investigated conditions, consistent with elemental balance analysis and the H_2_/CO ratio evaluation. Acetol reaction pathways do not change significantly as the temperature increases. The ROP analysis for H_2_O and CO_2_ shows that the RWGS reaction is active above 1100 K. Also, the reactions CH_4_ + OH = CH_3_ + H_2_O and H_2_ + OH = H + H_2_O contribute to H_2_O formation; however, the calculated H_2_O concentration remains negligible (<1%) within the considered temperature range. The total CO_2_ ROP becomes negative above 1200 K, marking the onset of CO_2_ consumption, via the reactions CO_2_ + H = CO + OH and CO_2_ + H_2_ = CO + H_2_O.

The addition of phenol introduces a parallel aromatic reaction pathway alongside the acetol decomposition chemistry, which remains substantially unaffected by the presence of aromatic species. Indeed, simulations suggest acetol is fully converted at 1000 K, while phenol decomposition starts for *T* > 1000 K. Phenol reacts according to the following reactions

$$C_{6}H_{5}{OH=CYC}_{5}H_{6}+CO$$

$$C_{6}H_{5}{OH+H=C}_{6}H_{6}+OH$$

$$C_{6}H_{5}{OH+H=C}_{6}H_{5}{O+H}_{2}$$

The reaction pathways of these product species lead to the formation of a broader spectrum of aromatic intermediates with respect to acetol, including benzene, benzofuran, naphthalene, and toluene, however, at very low concentrations (less than 0.015%). Soot precursors BIN1B and BIN1A reach 0.04% and 0.016%, respectively, at the highest temperatures, slightly higher than for pure acetol but still negligible.

The H_2_O and CO_2_ ROP patterns are qualitatively identical with those of pure acetol. The main gas-phase products (H_2_, CO, and CH_4_) are mainly formed by the acetol reaction pathways; therefore, they are produced across the entire temperature range in approximately the proportion expected from the reduced acetol content in the mixture. This suggests that the addition of phenol has a marginal contribution to the gas-phase composition, while it is responsible for the different composition of the liquid phase, consistent with the experimental findings.

For furfural, simulations predict the highest formation of PAH species among the feeds investigated, consistent with GC–MS identification.

According to reaction path analysis, the furfural ring releases H_2_ and CO by reacting with H radicals following the reactions C_5_H_4_O_2_ + H = H_2_ + C_4_H_3_O + CO and C_5_H_4_O_2_ + H = H_2_ + 2CO + C_3_H_3_. C_4_H_3_O is then converted into the following products

$$C_{4}H_{3}{O=CH}_{2}{CO+C}_{2}H$$

$$C_{4}H_{3}{O=CO+C}_{3}H_{3}$$

$$C_{4}H_{3}{O=HCCO+C}_{2}H_{2}$$

HCCO is then mainly converted into CH_2_CO, which finally produces CH_3_ and CO; instead, the other intermediates (C_2_H, C_2_H_2_, and C_3_H_3_) are involved in the formation pathways of aromatic species and PAH. Nevertheless, within the considered temperature range, the concentration of such species is still very low (C_6_H_6_, peak at 0.36%; C_6_H_5_C_2_H, peak at 0.028%; naphthalene, peak at 0.045%) and soot precursors BIN1B and BIN1A concentrations remain below 0.1%, with peaks above 1350 K, beyond the experimental window of interest. These results suggest that PAH formation has marginal relevance at the temperatures where dry reforming is the dominant process.

The H_2_O formation mechanism for furfural is negligible for temperatures lower than 1200 K, while for higher temperatures, it is still related to the RWGS reaction. The dominant CO_2_ consumption pathway at intermediate temperatures (below 1200 K) involves direct reactions with carbene intermediates, specifically CH_2_ + CO_2_ = CO + CH_2_O and CH_2_S + CO_2_ = CH_2_O + CO, which are generated by decomposition of CH_2_CO. Above 1200 K, the RWGS becomes the dominant CO_2_ consumption pathway for both feeds, with CO_2_ + H = CO + OH contributing as a secondary pathway.

The addition of syringol to furfural introduces the chemistry of the methoxy group (–OCH_3_). Syringol reacts with the H radical via three principal channels

$$C_{8}H_{10}O_{3}{+H=H}_{2}{+CO+CH}_{2}{CO+CH}_{3}{O+0.5C}_{4}H_{4}{+C}_{2}H_{2}$$

$$C_{8}H_{10}O_{3}{+H=H}_{2}{+CO+CH}_{3}{O+C}_{2}H_{2}{+0.5AC}_{3}H_{4}{+0.5PC}_{3}H_{4}$$

$$C_{8}H_{10}O_{3}{+H=0.5C}_{8}H_{10}O_{3}{+0.5C}_{6}H_{5}{OH+CH}_{3}O$$

C_4_H_4_, PC_3_H_4_, and AC_3_H_4_ are mostly converted to C_2_H_2_, which promotes the PAH growth. The PAH profile mirrors that of furfural, with BZFUR concentration slightly higher than for pure furfural (0.024% vs 0.018%). The H_2_O and CO_2_ ROP patterns are similar to those of the pure furfural.

The simulations suggest that the reactivity trends can be linked to the functional group composition of each feed. For acetol, thermal decomposition proceeds primarily through the break of the C–C bond between the carbonyl carbon and the hydroxymethyl group (–CH_2_OH), yielding acetaldehyde (CH_3_CHO) and formaldehyde (CH_2_O) as primary intermediates. This preferential bond breaking is a direct consequence of the bifunctional nature of the acetol molecule since the simultaneous presence of the ketone carbonyl (C=O) and the hydroxyl group (–OH) weakens the adjacent C–C bond.^37^ The resulting intermediates CH_3_CHO and CH_2_O are directly converted to CO, H_2_, and CH_4_, explaining the higher concentration of such species observed for acetol within the considered temperature range.

The aromatic hydroxyl group of phenol confers significantly higher thermal stability compared to the aliphatic hydroxyl of acetol. Phenol decomposition proceeds through three principal channels: direct unimolecular decomposition to cyclopentadiene (CYC5H6) and CO; substitution with H radicals to yield benzene and OH; and H-abstraction to form the phenoxy radical (C_6_H_5_O) and H_2_. The first pathway reflects the tendency of phenolic species to expel CO from the ring upon decomposition,^37^ while the second and third channels require the availability of H radicals and therefore become significant only above 1000 K, when the radical pool is sufficiently large. All three pathways lead to aromatic intermediates that contribute to the broader PAH spectrum observed in the condensed liquids of the acetol/phenol mixture without interfering significantly with the main gas-phase decomposition chemistry of acetol.

For furfural, the absence of free hydroxyl groups fundamentally changes the decomposition mechanism: the furfural ring reacts with H radicals generating C_4_H_3_O, C_3_H_3_, C_2_H_2_, and C_2_H that are key intermediates for PAH growth via the HACA mechanism, explaining also the richest aromatic spectrum, fully consistent with the naphthalene, biphenylene, and pyrene identified by GC–MS in the condensed phase.

The addition of syringol to furfural introduces additional reaction pathways associated with the methoxy group (–OCH_3_) whose C–O bond is relatively weaker, making methoxy cleavage the preferential initiation reaction for syringol, generating methoxy radicals (CH_3_O) that rapidly decompose to formaldehyde and H. In addition, syringol carries an aromatic ring with two methoxy substituents and a hydroxyl group, whose decomposition generates a more complex reactivity of the furfural/syringol mixture compared to pure furfural.

The onset and extent of CO_2_ conversion at high temperature can be directly linked to the feed reactivity through the availability of H radicals and H_2_, which the kinetic analysis identifies as the primary drivers of CO_2_ consumption via CO_2_ + H = CO + H_2_ and CO_2_ + H_2_ = CO + H_2_O, respectively, across all feeds investigated.

For acetol and acetol/phenol mixtures, the main sources of H radicals are the reactions HCO + M = CO + H + M and CH_3_ + H_2_ = CH_4_ + H that, along with the H_2_ formation from the feed decomposition pathways, activate the CO_2_ consumption at *T* > 1150 K.

For furfural and furfural/syringol mixtures, the reactions HCO + M = CO + H + M and HCCO + H_2_ = CH_2_CO + H are responsible for the formation of the radical pool and promote the CO_2_ conversion for *T* > 1170 K for furfural and *T* > 1220 K for furfural/syringol.

In general, the activation temperature of the dry reforming is comparable among the considered feeds; however, the extent of CO_2_ conversion is significantly lower for furfural-based systems compared with acetol-based ones, consistent with the different reactivities of the feeds and different levels of H_2_ and H radicals.

## 4 Conclusions

The dry reforming of bio-oils represents a promising strategy for carbon capture and valuable gas production with the potential to contribute to CO_2_ emission reduction. In this study, for the first time, this potential was investigated through experimental tests conducted on representative bio-oil components, selected based on a comprehensive literature review, in order to examine the behavior of the feedstock/CO_2_ system in terms of hydrogen yield, CO production, and the degree of CO_2_ conversion. In particular, acetol, phenol, furfural, and syringol and their mixtures were selected, and hydrocarbons like propane and methane were also considered as benchmarks. The behavior of the stoichiometric compound/CO_2_ mixtures was tested as a function of the temperature from 800 K up to 1350 K. The results show that the process evolved through two stages. At low temperature, the feed is mainly converted by thermal decomposition, forming H_2_, CO, CH_4_, small hydrocarbons (C_2_H_2_, C_2_H_4_), and heavier species, collected in the condensed phase. At higher temperatures, dry reforming is activated, as suggested by the sharp decrease of the CO_2_ concentration and reduction of CH_4_ and C_2_Hx species, while H_2_ and CO show a more pronounced increase.

The thermal dry reforming is activated at above 1300 K for methane, while the identified bio-oil components and propane were shown to be effectively converted into syngas via the dry reforming process at lower temperatures, in particular above 1200 K. This activation temperature remains essentially unchanged when considering pure compounds or mixtures of different compounds, while some variations are observed in the hydrogen yield, maximum CO_2_ conversion, and H_2_/CO ratio.

CO_2_ conversion of more than 50% was achieved, with values up to 100% for acetol and acetol/phenol mixture and for propane. Acetol and acetol/phenol mixtures also exhibit the highest hydrogen yields, approaching 100% at the highest temperatures, while furfural shows a lower reactivity. The addition of syringol to furfural enhances hydrogen production, particularly at elevated temperatures. However, it leads to a decrease in CO_2_ conversion, suggesting that the syringol decomposition pathways may partially compete with the activation of the dry reforming reaction. The evaluation of the experimental H_2_/CO confirms that, in the case of acetol-based and furfural systems, the high-temperature chemistry is controlled by the dry reforming reaction since the H_2_/CO is close to the ratio to the theoretical stoichiometric value (based on the dry reforming reactions). Conversely, for the furfural-syringol mixture, at high temperature, H_2_/CO is higher than the theoretical stoichiometric value.

Overall, the results demonstrate that bio-oil model compounds can be effectively converted into syngas via noncatalytic dry reforming at lower activation temperatures than methane, with CO_2_ conversion levels and hydrogen yields that are dependent on the molecular structure of the feed. Complete CO_2_ conversion and hydrogen yields approaching 100% were achieved for acetol-based feeds, while furfural-based systems showed lower but still significant performance, limited by the lower reactivity of the aromatic ring.

The influence of operating conditions such as residence time, the mixture composition, and the feed/CO_2_ ratio deserves further attention.

## Supplementary Material



## Notes

Residence time distribution and velocity profiles of the helical tubular flow reactor; gas phase species concentration for the dry reforming experiments; detailed GC–MS and KF analysis results of the liquid products collected from dry reforming; and simulated species profiles (PDF)
