# Supplementary material for: Thermal Dry Reforming of Bio-Oil Model Compounds
Source: Energy Fuels. 2026 Jun 23;40(26):14024–35. doi: 10.1021/acs.energyfuels.6c01305 (PMC13340431; doi:10.1021/acs.energyfuels.6c01305)
Supplement: Supplementary file 1 [file ef6c01305_si_001.pdf]

Supporting Information

Thermal Dry Reforming of Bio-Oil Model Compounds

Maria Virginia Manna, Davide Amato, Giovanni Fabozzi, Giovanni Battista Ariemma, Pino Sabia, Raffaele Ragucci, Mara de Joannon*

Institute of Sciences and Technologies for Sustainable Energy and Mobilities, STEMS-CNR, Napoli, Italy

*mariavirginia.manna@stems.cnr.it

1. Residence time distribution and velocity profile of the helical tubular flow reactor

The flow field inside the helical tubular reactor was computed by CFD simulations performed in ANSYS Fluent at two representative temperatures (900 K and 1350 K), using the experimental gas mixture (92% N_2-8% CO_2 by volume) modeled as an ideal gas with temperature-dependent transport properties. The simulations were carried out at the experimental mean velocity of 1.7 m/s.

Figure S1 shows the simulated velocity profiles along two orthogonal cross-sectional diameters (horizontal and vertical) at a representative axial location within the helical section, compared with the ideal parabolic profile at the same mean velocity.

At 900 K, the CFD profiles show a moderate deviation from the ideal parabola, with the velocity peak slightly shifted toward the outer wall of the coil due to centrifugal effects, consistent with the Dean number at this condition (≈37). At 1350 K, both profiles are more symmetric and closer to the parabolic ideal, reflecting the lower Dean number (≈22).

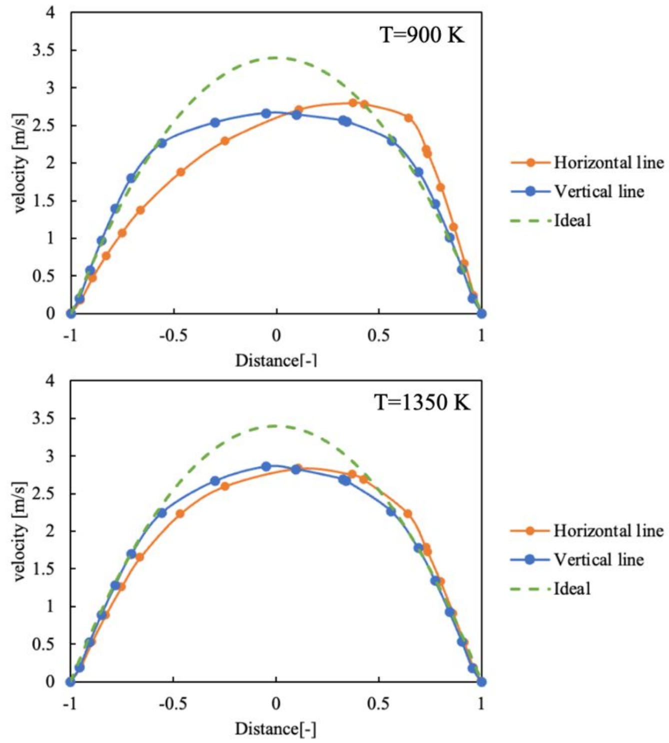

Figure S1. Simulated velocity profiles along two orthogonal cross-sectional diameters.

The residence time distribution (RTD) was characterized by a passive scalar pulse-tracer simulation at both temperatures (Figure S2). The outlet tracer response curves show a single well-defined peak centered at tau=3.1 s, in good agreement with the theoretical mean residence time. The RTD at 1350 K is slightly broader than at 900 K, consistent with the lower Dean number and correspondingly reduced radial mixing from secondary flows. Overall, the CFD characterization confirms that the reactor operates in the laminar flow regime under all experimental conditions and

approximates a straight laminar flow tube, with deviations from the ideal parabolic profile that decrease with increasing temperature.

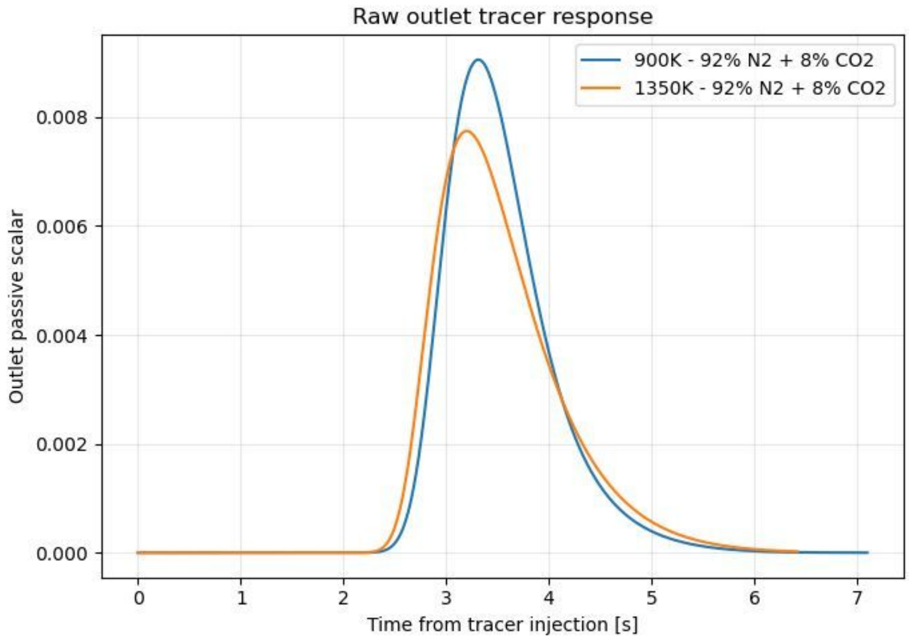

Figure S2. Simulated residence time distribution.

2. Complementary experimental data

Experimental concentration of C_2H_4 and C_2H_2 as a function of the temperature for different compounds. For furfural, furfural/syringol mixture, methane and propane, no significant amount C_2H_2 was detected. In case of methane, also the C_2H_4 concentration was negligible.

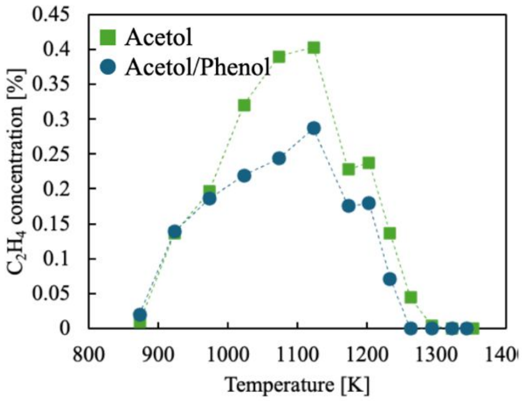

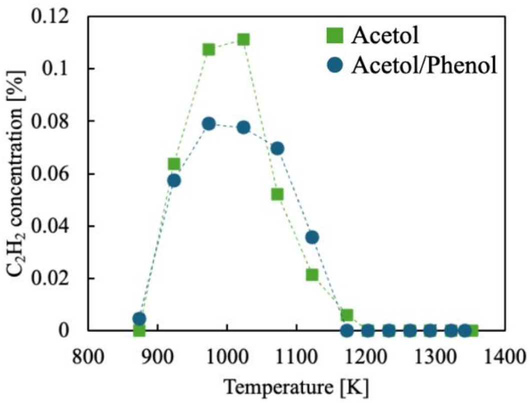

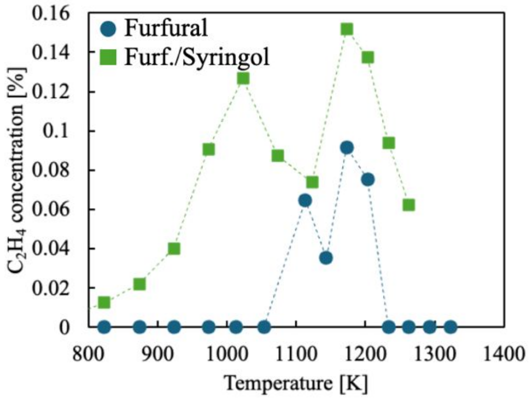

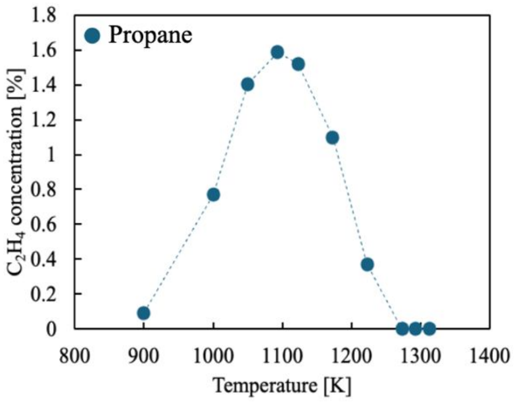

Acetol					
			823-923 K	*923-1073 K*	*>1073 K*
Compound	*Class*	*RT*	*Area %*		

Acetol	Hydroxyketones	10.9	92.29	91.66	87.25
Acetoin	Hydroxyketones	13.2	-	1.17	3.98
2-Pentanone, 4-hydroxy-4-methyl-	Hydroxyketones	21.1	2.40	1.12	1.16
			wt. %		
Water		-	5.3	9.3	11.9
Acetol-phenol					
			823-923 K	*923-1073 K*	*>1073 K*
Compound	*Class*	*RT*	*Area %*		
Benzene	Aromatic	8.4	0.19	0.15	0.11
Acetol	Hydroxyketones	10.9	54.83	9.05	4.09
Acetoin	Hydroxyketones	13.2	1.06	1.28	0.47
2-Pentanone, 4-hydroxy-4-methyl-	Hydroxyketones	21.1	0.79	0.47	0.38
Phenol	Phenolic	32.0	37.89	83.54	88.41
Acetic acid, phenyl ester	Phenolic	32.7	0.22	0.90	0.98
Phenol, 2-methyl-	Phenolic	35.0	-	1.86	2.88
p-Cresol	Phenolic	36.5	-	1.09	1.85
Naphthalene	Aromatic	38.9	-	-	0.18
Pyrene	Aromatic	81.1	0.59	0.36	0.28
			wt. %		
Water		-	4.9	8.9	10.0

Table S1 - Detailed GC-MS and KF analysis of the liquid products collected during the reforming of acetol and acetol-phenol mixture

Furfural					
			823-923 K	***923-1073 K***	***>1073 K***
Compound	***Class***	***RT [min]***	***Area %***		
Furfural	Furan	20.3	90.10	49.16	-
Benzofuran	Heterocyclic	28.4	-	0.22	-
2H-Pyran-2-one	Furan	29.9	4.25	11.39	-
2,2'-Bifuran	Furan	30.7	-	0.55	-
Indene	Aromatic	30.9	-	0.78	-
Phenol	Phenolic	32.0	-	0.45	-
p-Cresol	Phenolic	35.0	-	0.20	-
Naphthalene	Aromatic	38.8	1.93	13.94	-
2-ethenyl-Benzofuran	Heterocyclic	40.9	-	0.21	-
1-methyl-Naphthalene	Aromatic	44.3	-	0.99	-
2-methyl-Naphthalene	Aromatic	45.1	-	1.03	-
Biphenyl	Aromatic	47.9	-	1.54	-
2-Coumaranone	Heterocyclic	49.2	-	0.48	-
2-ethenyl-Naphthalene	Aromatic	50.6	-	0.79	-
Biphenylene	Aromatic	51.9	1.27	8.80	-
Fluorene	Aromatic	57.2	-	1.60	-
Anthracene	Aromatic	65.2	-	4.39	-
Phenanthrene	Aromatic	65.6	0.86	0.91	-

Pyrene	Aromatic	81.1	0.69	1.99	-
			wt. %		
Water		-	0.3	0.8	-

Table S2 - Detailed GC-MS and KF analysis of the liquid products collected during the reforming of furfural.

3. Simulated species profiles

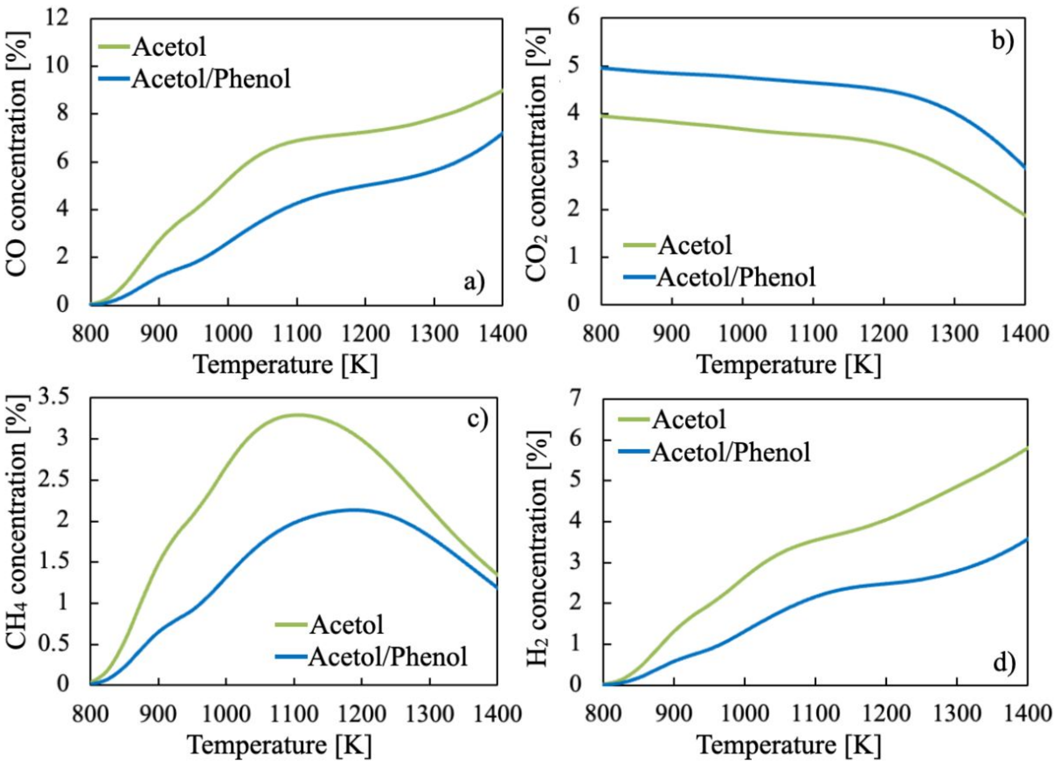

Figure S4. Simulated species concentration for acetol and acetol/phenol with Debiagi kinetic model.

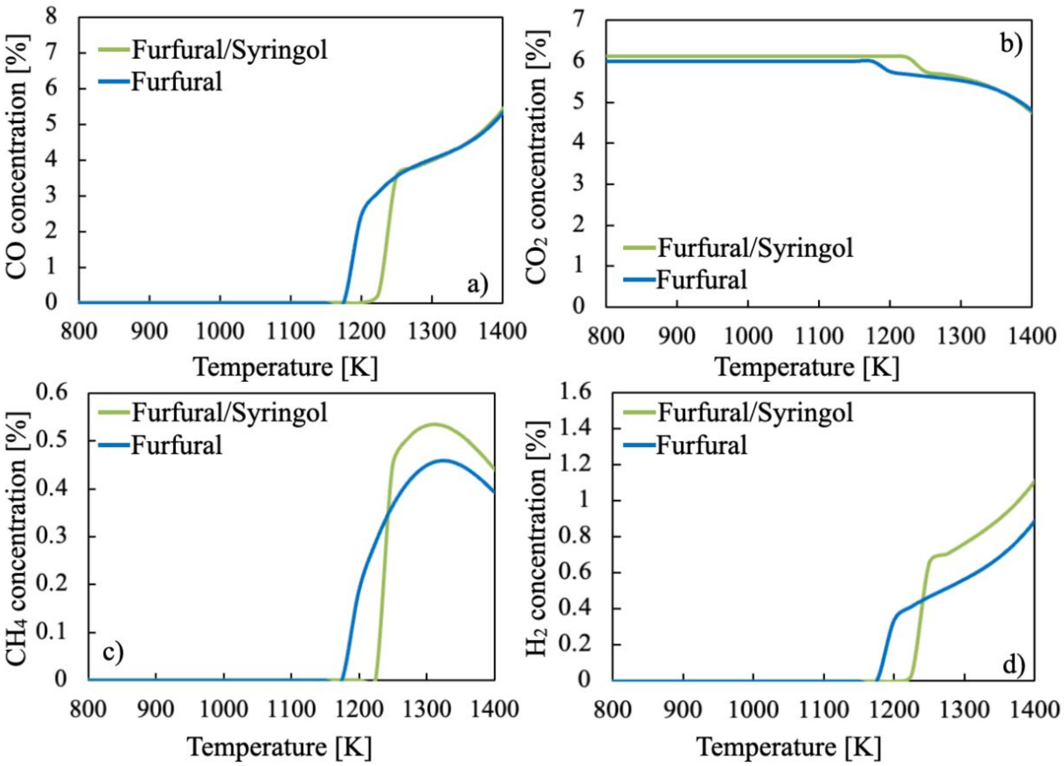

Figure S5. Simulated species concentration for furfural and furfural/syringol with Debiagi kinetic model.
